# Nonequilibrium thermodynamics and energy efficiency in weight loss diets

**DOI:** 10.1186/1742-4682-4-27

**Published:** 2007-07-30

**Authors:** Richard D Feinman, Eugene J Fine

**Affiliations:** 1 Department of Biochemistry, State University of New York Downstate Medical Center, Brooklyn, NY 11203, USA; 2 Department of Nuclear Medicine, Albert Einstein College of Medicine, Bronx, NY 10461, USA

## Abstract

Carbohydrate restriction as a strategy for control of obesity is based on two effects: a behavioral effect, spontaneous reduction in caloric intake and a metabolic effect, an apparent reduction in energy efficiency, greater weight loss per calorie consumed. Variable energy efficiency is established in many contexts (hormonal imbalance, weight regain and knock-out experiments in animal models), but in the area of the effect of macronutrient composition on weight loss, controversy remains. Resistance to the idea comes from a perception that variable weight loss on isocaloric diets would somehow violate the laws of thermodynamics, that is, only caloric intake is important ("a calorie is a calorie"). Previous explanations of how the phenomenon occurs, based on equilibrium thermodynamics, emphasized the inefficiencies introduced by substrate cycling and requirements for increased gluconeogenesis. Living systems, however, are maintained far from equilibrium, and metabolism is controlled by the regulation of the rates of enzymatic reactions. The principles of nonequilibrium thermodynamics which emphasize kinetic fluxes as well as thermodynamic forces should therefore also be considered.

Here we review the principles of nonequilibrium thermodynamics and provide an approach to the problem of maintenance and change in body mass by recasting the problem of TAG accumulation and breakdown in the adipocyte in the language of nonequilibrium thermodynamics. We describe adipocyte physiology in terms of cycling between an efficient storage mode and a dissipative mode. Experimentally, this is measured in the rate of fatty acid flux and fatty acid oxidation. Hormonal levels controlled by changes in dietary carbohydrate regulate the relative contributions of the efficient and dissipative parts of the cycle. While no experiment exists that measures all relevant variables, the model is supported by evidence in the literature that 1) dietary carbohydrate, via its effect on hormone levels controls fatty acid flux and oxidation, 2) the rate of lipolysis is a primary target of insulin, postprandial, and 3) chronic carbohydrate-restricted diets reduce the levels of plasma TAG in response to a single meal.

In summary, we propose that, in isocaloric diets of different macronutrient composition, there is variable flux of stored TAG controlled by the kinetic effects of insulin and other hormones. Because the fatty acid-TAG cycle never comes to equilibrium, net gain or loss is possible. The greater weight loss on carbohydrate restricted diets, popularly referred to as metabolic advantage can thus be understood in terms of the principles of nonequilibrium thermodynamics and is a consequence of the dynamic nature of bioenergetics where it is important to consider kinetic as well as thermodynamic variables.

## Background

Dietary carbohydrate provides both an energy source and, through its effects on insulin and other hormones, regulatory control of metabolism. In the context of obesity, diabetes and related pathologic states, it is argued by many researchers that the level of carbohydrate, by its hormonal effects, controls the disposition of nutrient intake beyond simple caloric balance [1-11]. From this point of view, fat plays a relatively passive role and the deleterious effects of high dietary fat are expected only if there is sufficient dietary carbohydrate to provide the hormonal state in which the fat will be stored rather than oxidized. In its practical application, the principle has given rise to several forms of popular diet strategies which have in common some degree of carbohydrate restriction [12-14] or effective glycemic level [3,15]. Experimentally, protocols based on carbohydrate restriction do as well or better than fat reduction for weight loss (reviews: [16-18]), but because they are somewhat iconoclastic with respect to official dietary recommendations and because they derive from the popular diets where discourse is heated, they remain controversial. The extent to which carbohydrate restriction is successful as a strategy for control of obesity or diabetes can be attributed to two effects. The strategy frequently leads to a behavioral effect, a spontaneous reduction in caloric intake as seen in *ad lib *comparisons. There is also a metabolic effect, an apparent reduction in energy efficiency seen in isocaloric comparisons, popularly referred to as metabolic advantage. The two are not necessarily independent: an association between thermogenesis, a reflection of inefficiency, and satiety has been established by Westerterp, et al., for example [19].

Experimental demonstrations of energy inefficiency in humans have recently been summarized [16,17,20] and the phenomenon has been demonstrated in animal models (e.g., ref. [21] and, most dramatically ref. [22]). This metabolic effect, however, is not universally accepted as a major component in human experiments, oddly even by investigators who have provided experimental support [23-26]. Variable energy efficiency, however, is known in many contexts: hormonal imbalance [27,28], intensive insulin therapy [29], studies of weight regain [30,31] and particularly knock-out experiments in animals [32-34]. Experiments demonstrating variable energy efficiency in the context of weight loss, however, remain controversial because of the difficulty in validating compliance in dietary interventions and because of a resistance to what is perceived as a violation of thermodynamics, that is, an intuitive feeling that, in the end, everything must even out. Thus, progress in this field still depends on a proper understanding of caloric efficiency and a description of how energy balance can account for differences in weight loss in isocaloric comparisons.

We have previously described how different isocaloric diets are actually expected to have different effects on metabolism and therefore on body mass [16,35,36]. Our previous arguments were largely based on equilibrium thermodynamics because this is most familiar. However, living systems, and in particular, TAG stores in adipocytes, are maintained far from equilibrium and the rates of breakdown of such high energy compounds are regulated by the kinetics of the enzymes that catalyze hydrolysis and re-synthesis. Because the system is maintained far from equilibrium, energy measurements provide values of (∂G/∂ξ)_T,P _where ξ is the reaction progress coordinate and the path-independence of state variables, that is, ΔG values measured in a calorimeter do not necessarily apply [37]. In essence, then, the problem is as much one of rates as of free energy. Much progress has been made in the development of nonequilibrium thermodynamics for the study of metabolism although there is no universally accepted approach ([38-40] and references therein) and the current work is intended to provide a first step towards developing the problem of energy efficiency in response to dietary macronutrients.

Here we review the basic ideas of nonequilibrium thermodynamics and provide an approach to the problem of maintenance and change in body mass following these ideas. The emphasis is on flux of metabolites in adipose tissue since, in the end, this is the major reflection of energy balance and obesity. The work has several goals:

1. To recast the problem of TAG accumulation and breakdown in the adipocyte in the language of nonequilibrium thermodynamics. In particular, we want to describe adipocyte physiology in terms of cycling between an efficient storage mode and a dissipative mode. Experimentally, this is reflected in the rate of fatty acid flux and fatty acid oxidation.

2. To provide a plausible mechanism for how different efficiencies of isocaloric diets can be accounted for by changes in kinetics. To show that hormonal levels controlled by changes in carbohydrate intake determine the relative contributions of the efficient and dissipative parts of the TAG-FA cycle.

Overall, the model is intended to provide a conceptual framework for energy efficiency in nutrition and to point the way to future research. We feel that the approach has general implications as well and is tied to the philosophical position espoused by Prigogine and followers in emphasizing the dynamic nature of physical processes, that is, the need to consider kinetics as well as thermodynamics [39,41-44].

We emphasize that metabolic efficiency is not always seen in diet comparisons. A thermodynamic analysis, however, shows that inefficiency is to be expected and it is the cases where "a calorie *is *a calorie" that need to be explained: it is the unique characteristics of living systems – maintenance of a steady-state through tightly controlled feed-back systems – not general physical laws that accounts for energy balance when it is found. Practically speaking, the importance of obesity and other metabolic disorders makes it important to see what the requirements are to break out of these stable states.

### Nonequilibrium thermodynamics

It is traditional to separate thermodynamics and kinetics but such a division applies strictly only to equilibrium systems [41,45]. Systems that are far from equilibrium may undergo chemical reactions that never attain equilibrium and are characterized by the flux of material as well as energy. In a dietary intervention, the flux of material must be integrated over time to determine the total change in weight or fat loss. Thus, accumulated changes may be controlled by the presence of a catalyst or other factors that affect the rate of reaction.

**Figure 1:**
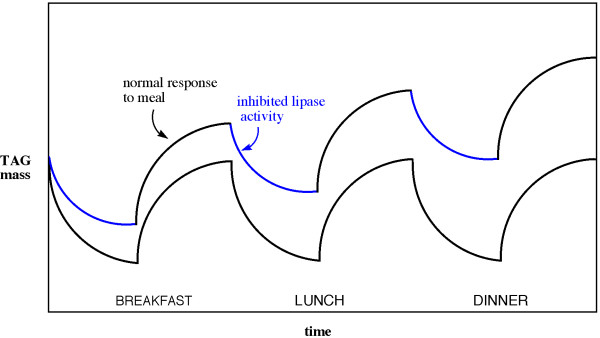
**Hypothetical kinetics of fat storage and hydrolysis**. Model for the effect of insulin on efficiency of storage. Black line indicates response under conditions of weight maintenance. Blue line shows the effect of added insulin on hormone sensitive lipase activity.

In the case at hand, adipocytes cycle between states of greater or lower net breakdown of fat (lipolysis and reesterification) depending on the hormonal state which, in turn, is dependent on the macronutrient composition of the diet. A hypothetical scheme for changes in adipocyte TAG and a proposal for how TAG gain or loss could be different for isocaloric diets with different levels of insulin is shown in Figure 1. Under normal control conditions of weight maintenance, the breakdown and utilization of TAG by lipolysis and oxidation is balanced by the re-synthesis from food intake. Assuming, for simplicity. an instantaneous spike in food at meals, the curves represent the net flow of material (possibly through several TAG-FA cycles) within the adipocyte. In a coarse-grained analysis, the integral over time of the fluctuations between different states, measures the change in stored TAG in the time of a dietary experiment. The average is stable, that is, appears as weight maintenance. If now each meal is maintained at constant calories but there is an increase in the percentage of carbohydrate leading to higher insulin levels, the lipases may be reduced in activity (blue line in Figure 1). The rate of re-synthesis of TAG is less perturbed by the elevated insulin [46] and indeed may go the other way. The system may cycle between states, which, while they never come to equilibrium, have the net affect of producing changes in the direction of accumulation of TAG.

In carbohydrate restriction, the decrease in carbohydrate may be accompanied by an increase in dietary fat and the relative effect on rate of TAG accumulation due to disinhibition of lipolysis vs the effect of increased substrate will determine the efficiency. As noted below, experiments in the literature [47] show that after chronic exposure to a low carbohydrate diet (higher dietary TAG), the plasma levels of TAG following a high fat meal are reduced compared to controls. Of course, replacing dietary carbohydrate with dietary protein at constant lipid will be consistent with the model in the absence of compensating effects.

In these cases, the integrated change in TAG over the course of a day (or several days) will no longer be zero. In this way, two diets may lead to different weight gain (as indicated by accretion of fat), even though they have the same number of calories, simply because they affect hormonal levels differently. An analysis based on rates suggests further that a new steady state may be obtained in which TAG may be maintained at a higher or lower level even if the hormonal state returns to one that does not lead to further change. The cell may then relax from one steady state to another, the observed macroscopic weight gain or loss. The goal here is to ask what would it take to produce behavior like that in Figure 1.

For minor perturbations, there will be compensating effects of competing pathways (increase in insulin secretion due to fatty acid production [48,49], for example) and one can expect, insofar as the model corresponds to reality, there may be a threshold effect. This is reflected in the emphasis on extreme carbohydrate reduction in the early phases of popular weight loss diets [12-14]. We emphasize that all of the potential sources of metabolic inefficiency – increased reliance on gluconeogenesis and consequent increased protein turnover, up-regulation of uncoupling proteins – described previously [16,36] may still be operative but the net change in fat stores must be the final common output if body mass is to undergo change.

### Formalism of nonequilibrium thermodynamics

For systems that are not at equilibrium, changes in entropy will drive the system towards equilibrium. If the system is close to equilibrium or, as in the case here, there is a small change in the total free energy – only a small fraction of TAG is actually hydrolyzed in the course of a day – then the change in entropy will be due to dS_e_, the flux of entropy that is exchanged with the environment and dS_i_, that due to the irreversible effect of the chemical reaction [41,50,51]. We are then interested in the rate of entropy production, Φ, due to chemical reactions at constant T and P:

Φ = dS_i_/dt = - (1/T) Σ^N ^μ_k _dn_k_/dt

In nonequilibrium thermodynamics, overall flux of entropy is considered as a product of forces (derivative of the potential), X_k _and flows J_k_, all forces and flows vanishing at equilibrium. In a chemical system, the force X_k _is defined as the negative of the chemical potential of the kth reaction, sometimes referred to as the affinity A = - (∂G/∂ξ)_T,P _where ξ is the extent of chemical reaction. In other words, a positive sign of × indicates spontaneous forward driving force. The force, then, depends on the concentration of reactants and products, the standard free energy and the extent of reaction. It is worth noting that for the systems like the adipocyte that are maintained far from equilibrium the distinction between ΔG values and (∂G/∂ξ)_T,P _noted by other authors [37] is important, that is, the simple additivity of state variables that underlies the idea that all calories are equivalent, is not valid.

The flows, J_k_, are identified with the flux of the kth reaction. The flux of fatty acid in an adipocyte, for example, J_1 _= v_lipolysis _+ v_synthesis_, the sum of breakdown and synthesis rates for TAG. In the phenomenologic approach of nonequilibrium thermodynamics, the forces and flows may be the sum of several individual processes.

In applying the principles of nonequilibrium thermodynamics, the analysis will be simplified if we make the assumption that the fluxes are linear functions of the forces, in analogy with similar linear equations such as Fick's law of diffusion (diffusion is a linear function of the concentration gradient), or Ohm's law (current is a linear function of the potential). The proportionality constant L_kj _is called the phenomenological coefficient.

J_k _= Σ^n ^L_kj_X_j _

Although the general requirement that condition (2) hold is that the system be close to equilibrium, the linear approximation is often observed to be appropriate for systems very far from equilibrium, subject to stabilizing feedback and in enzymatic systems operating in the range of substrate concentrations that are close to K_M _[52,53]. Further discussion is found in references [54-56]. Whereas the assumption of linearity is reasonable for the current model where small perturbations far from equilibrium occur in a region of high substrate, in the end, it is a working assumption and experimental tests of the model will ultimately determine if the assumption is justified.

### Qualitative features of the adipocyte model and comparison to glycolysis

F igure 2 shows a simple model that is proposed for adipose tissue metabolism under conditions bearing on changes in body mass. The flux of TAG (1) represents the net accumulation or output with respect to the cell itself. This process driven by (2) the input of glycerol-3-phosphate from glycolysis or glyceroneogenesis and (3) fatty acid (FA) from plasma FA. The high energy form of the cycle, TAG, is stored. From the point of view of the organism, it is the FA output that provides fuel for oxidation and cell metabolism. This output may be taken as analogous to the system load as it is usually described in nonequilibrium thermodynamics. Oxidation and FA uptake are largely controlled independently, that is, the adipocyte system has high output conductance and low input conductance, that is, by analogy with an electronic system, is an ideal amplifier. Because there is effectively no load on the system and overall metabolic effect is simply to reduce the affinity of fatty acid, the analysis is greatly simplified. The flux of FA, J_3 _is of general physiologic importance and is the most experimentally accessible of the relevant parameters.

In the comparison of different diets, an additional component is (4) input of fatty acid from TAG-containing lipoproteins. Our treatment of the problem is to consider fluxes in the absence of this input since that is how it is usually described in the literature and then to consider the effect of input from lipoproteins as a perturbation. Focusing on the reaction in the absence of lipoprotein input, the overall relations of fluxes and flows:

J_1 _= L_11_X_1 _+ L_12_X_2 _

J_2 _= L_21_X_1 _+ L_22_X_2 _

J_3 _= L_13_X_1 _+ L_33_X_3 _

As an example of the application of these principles, Aledo, et al. addressed the negative correlation between glycolytic flux and intracellular ATP concentration in yeast, the so-called ATP paradox [54,57,58]. The paradox was resolved by showing that if ATP-consuming pathways are more sensitive to glucose than the glycolytic pathway, the cell can switch from an efficient (ATP-conserving) to a dissipative (ATP-utilizing) regime [54,58]. The dissipative regime offers higher output at high glucose cost, whereas the efficient regime has higher accumulation of ATP but lower glycolytic flux.

In the adipocyte model, periodic switching between dissipative and conservative regimes is meant to describe the dynamic cycling of TAG. The goal in development of the model is to show the constraints on the system for conservation of fat mass, and conversely, how isocaloric dietary inputs of different composition might plausibly bring about weight gain or loss, that is, how efficiency is regulated in the TAG-FA cycle and the activity of the reactants. In essence, we want to know what it would take for the blue line in Figure 1 to occur.

The major controlling variables will be the L_ij_, the phenomenologic constants which depend on hormonal levels, and the thermodynamic activity of plasma triglyceride (supplying fatty acid). Looking ahead, the simplest application will be the effect of replacing dietary carbohydrate with dietary protein at constant lipid where a semi-quantitative prediction can be made. In the most general case, however, we also want to know the relative impact of insulin reduction on the L_ij _(reduced lipolysis rate) compared to the increase in thermodynamic activity (X_4_) due to increased dietary fat.

The variables as they apply to the adipocyte model are as follows:

X_1 _= the output force is the affinity of the lipolysis-TAG synthesis cycle. The analysis can be simplified by the assumption that lipolysis of available TAG (and possibly re-synthesis) in an adipocyte occurs at a heterogeneous interface. We can therefore take the thermodynamic activity of TAG as 1, that is, although other concentrations may influence X_1_, the amount of TAG will not. (The contribution of TAG activity is unlikely to change in any case since perturbations in TAG concentrations are extremely small compared to the total stored TAG).

X_1 _= -RT (ln (K_eq_)^FA-TAG ^- ln ([FA]^3 ^[glyc-3-P]/[TAG]) = - 3 RT ln (([FA] [glyc-3-P]/K')

X_2 _= the driving force for supply of glycerol-3-phosphate whose major term is normally the availability of carbohydrate. Under conditions of carbohydrate restriction, however, there is also an increase in glyceroneogenesis from protein [59,60].

X_3 _= = the driving force for supply of fatty acid from cellular TAG.

X_4 _= the force due to the supply of fatty acid from lipoproteins (chylomicrons and VLDL).

In the approach taken here,

L_11_, L_12 _are the sensitivities of the flux of TAG to the levels of TAG and the levels of substrate (glycerol-3-phosphate) which depend primarily on the hormonal levels (*via *phosphorylation of the lipases and other enzymes). It is generally assumed on theoretical grounds (Onsager relation) that L_12 _= L_21 _although this has to actually be established for systems that are not close to equilibrium.

L_22 _is the sensitivity of the glycerol-3-phosphate flux to the availability of carbohydrate (or other sources) which may also be controlled by hormonal levels.

**Figure 2:**
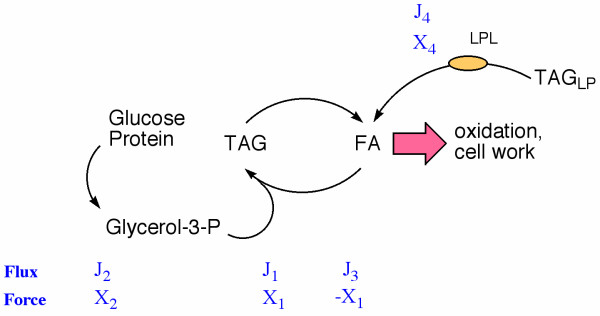
**Model for adipocyte metabolism**. See text for details.

Although somewhat beyond the level of analysis presented here, it is worth noting some of the derived parameter that are traditional in a NET analysis. The degree of coupling, q = L_12 _/√L_11_L_22 _is a dimensionless parameter that indicates how tightly the output process is coupled to the driver process [55] and takes on values from 0 to 1 in the forward direction. In the model in Figure 2, q will vary with different subjects and different metabolic states, in particular, is strongly under the control of insulin.

The phenomenological stoichiometry is defined as Z = (√L_11_/L_22_)

It should be noted that L_11_, L_12 _and L_22 _and the derived parameters, q and Z, in general, are where the enzymatic activity and the effect of hormones reside. It is important to emphasize that many important variables, such as coenzyme levels are hidden in the phenomenologic constants. For the adipocyte Z = 1, that is, TAG synthesis is tightly coupled to glycerol-3-P production. These parameters hold the promise to quantify insulin resistance, at least in the adipocyte.

The experimental parameters that are most frequently determined in the literature are the rates of appearance in the blood of fatty acid and glycerol, traditionally written R_a _(FA) and R_a _(glycerol), and the total rate of TAG oxidation (largely oxidation in non-adipocyte), denoted here as R_OX_. For the simple two compartment model considered here, there are four species, adipocyte TAG, plasma TAG, FA and CO_2 _(from oxidation).

The goal is to re-cast the problem of metabolism and regulation of body mass in the formalism of nonequilibrium thermodynamics, or more simply, in a way that emphasizes rates in addition to energetics. Applying the traditional measurements above leads to particularly simple form. From conservation of carbon mass of fatty acid species, we can write for the mass fluxes:

0 = d(FA)/dt + d(TAG)/dt + d(CO_2_)/dt = R_a _(FA) + J_1 _+ R_OX _+ J_4_

*or *J_1 _= - (R_OX _+ R_a _(FA) + J_4_)

J_3 _= R_a _(FA)

Although no experiment in the literature has been done that would allow for a complete quantitative test of the model, further analysis can support the value of a nonequilibrium approach in understanding variable efficiency in weight loss experiments. Experiments comparing the effect of different macronutrient composition, for example, can allow us to look at the effects on TAG accumulation (J_1_) without explicit analysis of the individual reactions. Results from the literature that support the underlying thesis show that 1) fatty acid flux and oxidation (R_OX _+ R_a _(FA), eq. (6)), follow the levels of dietary carbohydrate, 2) the effect of carbohydrate is expressed in the regulation of insulin levels, 3) lipolysis is the primary target of insulin, 4) the availability of substrate affects efficiency, 5) insulin increases J_4 _and finally, 6) chronic diet can affect the force X_4 _and thereby the response to dietary input in a single experiment. In the following sections, we consider these in turn. The net effect is that accumulated time-dependent changes due to carbohydrate intake control the efficiency of fat storage and we consider that a nonequilibrium thermodynamic approach allows clear justification as to how variable weight gain can be expected on isocaloric diets.

### Fatty acid flux and oxidation follow the levels of dietary carbohydrate

#### Similarity of starvation and carbohydrate restriction

Over the years, several investigators have made the observation that the metabolic response to carbohydrate restriction resembles the response to starvation, in particular, for the current model, increased fatty acid mobilization and oxidation [61-65]. Perhaps the best example is an elegant study by Klein & Wolfe [65] comparing responses of subjects on an 84 hour fast to the same subjects on a similar fast in which lipids were infused at a level equal to resting energy requirements. Table 1 shows that the levels of glucose, insulin, the rates of appearance of fatty acid and fat oxidation, R_OX _+ R_a _(FA), were similar in the two groups. For comparison to equation (6), the molar fluxes would have to be converted to mass and the role of J_4 _would have to be considered explicitly. In fact, as measured here, J_4 _is subsumed in the rate of fatty acid appearance and appears to have little effect despite large differences in X_4_. These rather dramatic results were summarized by the authors as demonstrating that "carbohydrate restriction, not the presence of a negative energy balance, is responsible for initiating the metabolic response to fasting." It might be said that this was the fundamental observation for understanding the role of carbohydrates in energy balance and the need for a kinetic rather than equilibrium thermodynamic analysis. The controlling variables are presumed to be carbohydrate itself which provides substrate for glycerol-3-P synthesis and insulin which will affect the phenomenologic constants. Bisschop, et al. [62] showed a similar increase in FA rate of appearance and oxidation in a low carbohydrate, high fat diet (CHO:Lipid:Protein = 2:83:15) compared to either a high carbohydrate (85:0:15) or control (44:41:15) diets, and there is agreement with Klein & Wolfe's data (Table 1). Considering the difference in protocol, the similarity of the response to carbohydrate restriction, fasting and fasting + lipid is very good. Although the subjects in Klein & Wolfe's study lost comparable amounts of weight in the two procedures, the short duration and the substantial changes in body water make it difficult to accurately determine whether TAG storage follows the calculated value of J_1 _[65]. It is important to point out that in Bisschop's experiment, fatty acid oxidation does not keep up with the increase in dietary TAG but according to equations (6) and (7), the flux of TAG is increasing in the direction of breakdown of TAG and, again, explicit inclusion of J_4 _would further bias the results in that direction.

**Table 1:** Similarity of the effects of starvation and carbohydrate restriction on fatty acid flux and oxidation

Study/subjects	Glucose	FA	R_ox_	R_a _FA	- (R_OX _+ R_a_FA)
	(mg/dl)	(μmol/l)		(μmol/kg/min)	

Ref: [62] High CHO	91.8	0.38	0.90	1.52	2.42
control	91.8	0.42	1.25	1.76	3.01
High Fat	77.4	0.75	1.83	2.98	4.81
Ref: [65] Fast 84 h	68	0.92	1.94	0.97	2.91
Fast + Lipid	66	1.02	1.67	1.00	2.67

Data from references [62] and [65].

Although it would obviously be difficult to carry out experiments for long periods of time in humans, studies by Tomé's group have shown that rats fed a high fat diet without carbohydrate ate less and also gained less weight per calorie consumed than rats fed a high fat diet that included carbohydrate [21]. Similar results have recently been published by Kennedy, et al. have shown that a high fat/ketogenic diet could reverse the obesity induced by an isocaloric high fat diet that also contained sucrose [22]. The principle that the level of dietary TAG plays a passive role and that carbohydrate restriction is controlling suggests that evidence from the older literature showing weight loss on very high fat diets [66] might be worth re-examining. These were presumably not followed up because they were so counter-intuitive.

#### Glucose flux regulates TAG flux

Wolfe and Peters [67] measured the response to infusions of glucose in humans. The data shown in Table 2 indicate that the flux of glucose regulates the rate of TAG synthesis largely through the inhibition of lipolysis. The effect of glucose, in turn, is presumed to rest primarily with the effect of insulin.

**Table 2:** The effect of glucose flux on calculated TAG flux

R_a_(glucose) (μmol/kg/min)	fat oxidation (μmol/kg/min)	R_a _FA (μmol/kg/min)	- SUM	Δ SUM
Basal	3.74	5.78	9.52	-
5.6	3.14	4.96	8.10	1.42
22.2	3.48	3.25	6.73	2.79
44.4	1.99	2.43	4.42	5.10

Data from reference [67].

### The effect of carbohydrate is expressed in the regulation of insulin levels

#### Lipolysis is the primary target of insulin

**Figure 3:**
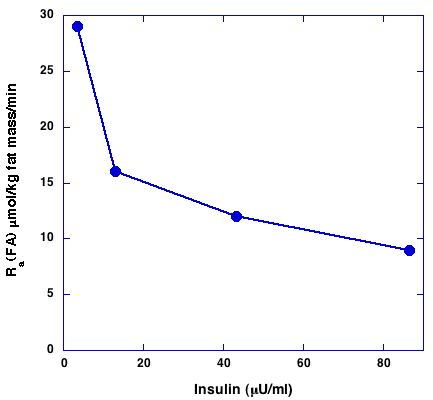
**Effect of insulin on fatty acid flux**. Free fatty acid appearance in plasma R(a) were examined in healthy humans infused intravenously with insulin. Data from reference [46]. Units converted for comparison to figure 5.

It is well established that the primary effect of insulin, both kinetically and in terms of physiologic effect is on the inhibition of lipolysis and there is a large literature studying this effect (Review: [68]). In the language of nonequilibrium thermodynamics, this is expressed in the phenomenologic constant, L_11_. Campbell, for example, studied fatty acid metabolism in humans infused intravenously with insulin [46]. Figure 3 shows the decline in fatty acid flux as the plasma insulin is increased. Oxidation of fatty acid was also inhibited but by a much smaller amount, from 2.7 to 0.9 μmol/kg lean body mass/min. The total rate of primary reesterification (from fatty acid that is not released to the plasma after lipolysis) was similarly increased. Insulin levels further increase the uptake of plasma TAG due to increase lipoprotein lipase (LPL) activity. Frayn and coworkers[69,70] have shown how the combination of LPL and lipolysis leads to increase in flux towards TAG storage. Again, the relative hormonal reduction in lipolysis and any increase in esterification due to mass action if plasma TAG is increased will determine if net TAG accumulation will occur. The importance of insulin can be seen in studies in which insulin secretion is indirectly inhibited via administration of a somatostatin antagonist octreotide. This intervention leads to a reduction in fat mass [6]. Conversely, it has long been known that chronic insulin therapy for diabetes leads to weight gain and decreased flux of fatty acids compared to isocaloric controls.

**Figure 4:**
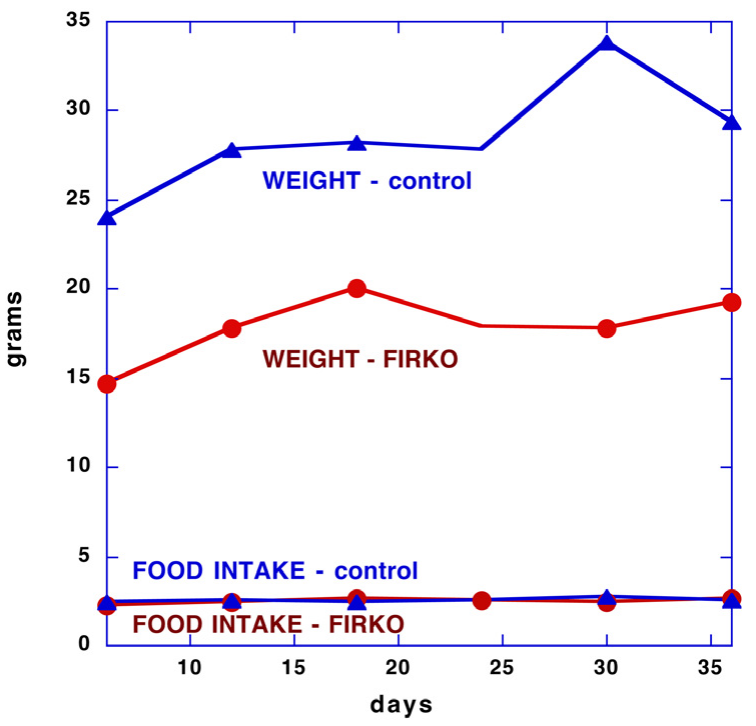
**Weight change and food intake of the FIRKO mouse**. Data from reference [71, 88] Adipose-specific insulin receptor knockout (FIRKO) mice have normal or increased food intake but are protected from obesity.

The most dramatic if abstract demonstration of the potential effect of carbohydrate restriction on insulin stimulation of fat cells comes from the study of the adipose-specific insulin receptor knockout mice FIRKO mouse of Bluher & Kahn [32,71]. These animals have a knockout of the insulin receptor specific to the adipocyte. Widely discussed because of their increased longevity they also show greatly reduced efficiency in the storage of lipid and are significantly thinner than the wild type even though both groups consumed the same amount of food (Figure 4).

#### Insulin Flux

**Figure 5:**
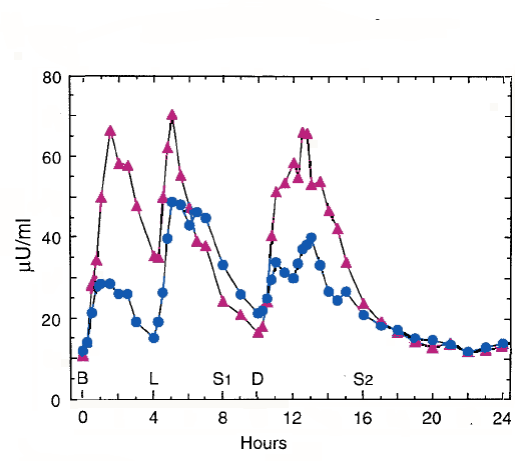
**Effect of diet on serum insulin concentration**. Mean serum insulin concentration before (red) and after (blue) 5 weeks on a reduced carbohydrate diet (CHO:Lipid:Protein = 20:50:30) using a randomized crossover design with a 5-week washout period. Data from reference [72]. The control diet was (55:30:15). As noted in the text, the insulin values are in the linear range of the dependence of fatty acid flux on insulin and the pattern roughly proportional to the flux of fatty acid.

The flux of insulin for diabetic patients under two dietary conditions is shown in Figure 5. A consistently lower level of insulin throughout the day is seen under conditions of lower carbohydrate intake. In addition, Such behavior has been measured frequently in the literature. Chronic carbohydrate restriction means that this reduced insulin never catches up with control. The study from Gannon & Nuttal [72] was carried out under conditions of weight maintenance so that there is presumably a compensating fatty acid oxidation but it is clear that insulin flux is controlled by dietary carbohydrate which, in turn, reduces the flux of fatty acid.

### Availability of substrate affects efficiency

#### Glycerol-3-phosphate: PEPCK overexpression

The key substrate for TAG synthesis is glycerol-3-phosphate. Because adipocytes normally have very low levels of glycerol kinase, the flux of TAG is dependent on processes (J_2_) that supply glycerol-3-phosphate: glycolysis or, under conditions of starvation or glucose deprivation, glyceroneogenesis, a truncated form of gluconeogenesis [59]. These processes are dependent on the composition of the diet and the hormonal state of the organism. One approach to separating the effect of glucose from the effect of glucose-induced insulin, is the genetic manipulation of the level of enzymes under conditions of low glucose. Such a strategy allows one to isolate the driving force from the effects of hormone on the phenomenologic constants, L_22 _and L_21_. Franckhauser [73] overexpressed phosphoenolpyruvate carboxykinase (PEPCK) in mice adipocytes. Under conditions of starvation, transgenic mice showed increased glyceroneogenesis which was accompanied by increased reesterification of free fatty acids (FAs), and a corresponding decrease in circulating FAs, both reflecting an increase in stored TAG (Table 3). In fact, the transgenic mice showed increased adipocyte size and fat mass, and higher body weight. Insulin sensitivity was preserved. When fed, nutrient consumption was the same for the experimental animals and the wild type. Thus, the change in the enzymatic activity of PEPCK affects the accretion of fat in the absence of any change in caloric intake or change in hormonal level that normally triggers changes in PEPCK levels. An overall change in the efficiency of food utilization is 2-fold for the heterozygotes and almost 4-fold for the homozygotes.

**Table 3:** Effect of overexpression of PEPCK of starved mice on feeding

Starved mice	PEPCK activity (%)	(J_2_) pyruvate -> glycerol (cpm/mg prot/2 hr)	(J_1_) FA reesterification (mmol/mg prot/2 hr)	J_1_/J_2 _(mmol/cpm)	FAT PAD Wt. (mg)	Food consumed mg ± SE
control	100	300	300	1.0	400	3.3 ± 0.1
hetero-PEPCK	400	650	550	1.18	800	3.2 ± 0.1
homo-PEPCK	1300	750	620	1.21	1500	3.4 ± 0.1

Data from reference [73].

In similar experiments, Shepherd, et al. [74] overexpressed adipocyte GLUT4 in transgenic mice. Body lipid was increased 2–3 fold in these mice compared to wild-type and the mutants had increased insulin sensitivity. Direct comparison to the simple model in Figure 2 is complicated by the fact that the transgenic mice showed fat cell hyperplasia rather than a simple increase in size.

#### Dietary fat and the effect of chronic carbohydrate restriction

**Figure 6:**
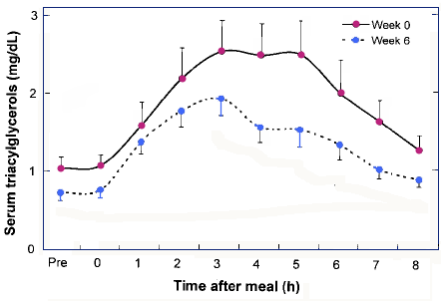
**Effect of chronic diets on postprandial response to high fat meal**. Responses to high fat meal before and after 6 weeks on low carbohydrate (< 10% energy) ketogenic diet in overweight men. Data from reference [47].

The key question in the application of the model is the extent to which lipolysis and other catabolic processes that are increased by reductions in insulin are compensated for by the increased availability of dietary TAG (X_4_) if carbohydrate in the diet is replaced by fat. At this point, we can consider the process indicated by J_4_, the influx of plasma FA from plasma TAG, as a perturbation on overall TAG storage. The activity of lipoprotein lipase (J_4_) is increased by higher insulin and will be reduced by chronic carbohydrate restriction [75]. The effect of chronic diet on the response to dietary fat challenge can provide further data on this point. Sharman, et al. [47] showed that six weeks on a low carbohydrate ketogenic diet led to a substantially reduced postprandial serum triacylglycerol (TAG) response in normal-weight men (Figure 6). The low carbohydrate group, in distinction to controls, showed drastically reduced (-34 %) insulin levels. Thus, despite the higher fat intake, the rate of lipolysis increased and the contribution of activity of TAG (X_4_) went down.

#### The bottom line: efficient and dissipative modes

While no experiment in the literature measures all the relevant variables, comparisons of Figures 3, 5 and 6 give a sense of the difference in time dependent responses on low carbohydrate and high carbohydrate (high insulin) diets. The individual components that contribute are as follows:

1. Rate of lipolysis. Insulin represses lipolysis as shown in Figure 3. This is true even in insulin-resistant states such as diabetes. Carbohydrate restriction reduces insulin fluxes as indicated in Figure 5.

2. Figure 6 shows that the effect of chronic carbohydrate restriction compared to controls is to reduce plasma triglycerides (X_4_) in response to a fat challenge, reducing the activity of FA in the carbohydrate-restricted state compared to the higher carbohydrate state.

3. Lipoprotein lipase is known to be up-regulated by higher insulin increasing the flux of FA into the adipocyte (J_4_) under conditions of high carbohydrate.

4. Carbohydrate represses per cent fat oxidation.

Thus, all of the differences in high and low insulin states are in the direction of efficient modes in the former and toward more dissipative modes in the latter.

As a guide to future research, then, the continuous monitoring of FA flux and oxidation, or other variables that allow determination of TAG flux can be done with current technology and it is possible to test the role of kinetic regulation in different weight loss strategies and to rationalize variable efficiency. It is important to point out that a thermodynamic analysis explains the potential for the metabolic advantage for particular diets but can, as well, point the way to identifying other factors that maintain homeostasis. In other words, isocaloric diets do not always show differences in efficiency and the thermodynamic analysis suggests that it is as important to explain cases where metabolic advantage does not occur as those where it does.

### Conductance matching

A metabolic scheme of the type considered here is traditionally evaluated in terms of the effect of the demand on the output by the load, or conductance matching by analogy with electronic systems [54,58]. The assumptions of the model in Figure 2 is that there is effectively no load on the adipocyte: output of fatty acid and its subsequent utilization by other tissues, are independently regulated and the adipocyte, in effect, has very high output conductance, that is, supplies whatever fatty acid is required. Wolfe and coworkers [76-78] have emphasized the extent to which glucose controls fatty acid metabolism rather than the other way around as originally suggested in the Randle cycle [79,80]. From the perspective of further metabolic analysis, the adipocyte may be considered a discrete modular element and could be patched into a larger network.

## Discussion

Variable metabolic efficiency due to the macronutrient composition of the diet is plausibly explained in terms of nonequilibrium thermodynamics by a shift in the cycling between dissipative lipolytic modes and efficient storage modes. Such a mechanism is consistent with experimental data on the effect of diet on metabolism. The nonequilibrium thermodynamic approach and the application to the FA-TAG cycle may raise general questions about metabolism.

### Fatty acid flux, insulin resistance

There is an increasing perception that circulating fatty acids are critical in metabolic responses and, in particular, in the development of insulin resistance and type 2 diabetes [81-83]. The effect of insulin resistance on the disinhibition of lipolysis and an increase in fatty acid flux may be as important for the adipocyte as the effect on glucose uptake. In combination, the two effects may reduce TAG storage and may represent a down-regulation in response to excess insulin. As such, it may be thought of as beneficial for obesity and, at the same time, suggests that reduction in insulin directly or via carbohydrate restriction will improve insulin resistance.

The increase in circulating fatty acid remains problematical in that, whereas it does indicate that less TAG is stored, it is generally considered deleterious and may lead to peripheral insulin resistance. In addition, fatty acids are known to stimulate insulin secretion. On the other hand, the effects of high plasma FA may be different under conditions of low carbohydrate: FA-induced insulin secretion, for example, is strongly dependent on carbohydrate levels [48] and is probably not a factor at all if plasma glucose is low. In practice, carbohydrate restriction improves insulin resistance and the increased fatty acids may be considered a reflection of a more general paradox: it is observed that fatty acid levels are increased in obesity[68] and references therein), diabetes and insulin resistance but are also elevated by those conditions that mediate against these conditions: exercise, starvation and carbohydrate restriction. It is also paradoxical that the TZD's increase insulin sensitivity but also pre-dispose to obesity. The latter effect has been shown to be due at least partly to the increase in glyceroneogenesis (X_2_) [59,84]. It could also be argued that the high levels indicate that FA is not being taken up by peripheral tissues as happens in insulin-resistant states. A recent review by Westman argues similarly that a so-called glycolytic pressure controls the disposition of fatty acid as fuel in muscles [85].

### General perspective

Animal models provide very clear-cut demonstrations of inefficiency as a function of macronutrient composition and therefore it seems there is no theoretical barrier to accepting demonstrations in humans where ideal control is not possible. The driving force for TAG flux in the proposed model is the availability of carbohydrate and the key regulating phenomenologic constant depends on insulin and other hormones. Of course, the system is going to be subject to other cells and processes. De novo fatty acid synthesis is a significant effect. Moreover, this simple model makes no attempt to account for compensatory processes and the nonlinear effects that are ultimately expected in complex biological systems. For example, hepatic production of β-hydroxybutyrate, which increases twenty-fold during very low carbohydrate diets, inhibits lipolysis [86], likely blunting the effects of reduced insulin concentrations.  The increased fatty acid flux under carbohydrate restriction will lead to increased insulin secretion and, at some point, these process would have to be added back into the model. 

### Relation to previous arguments on reduced energy efficiency

We previously pointed out a number of errors in the idea that weight regulation is necessarily independent of diet composition (and therefore insulin levels) [16,35,36]. We proposed several mechanisms and, in a practical sense, all of these – increased gluconeogenesis and associated increased protein turnover, increased mitochondrial uncoupling and increased substrate cycling – must be reflected in the flux of TAG if fat loss is to be effected. We have also pointed out that in a dietary intervention it is important to be specific about changes in fat mass not simply weight loss [87]. The mechanism is ultimately through fatty acid oxidation which, again, will be under separate control of glucose and hormones.

From a theoretical standpoint, the simplest objection to the idea that calorimeter values are sufficient to understand processing of food is that it assumes that no process other than complete oxidation takes place, that is, that metabolic reactions are the same as calorimeter reactions. This is obviously not generally true since living organisms use other reactants and make all kinds of products, proteins, ATP, etc. In comparing two diets of different macronutrient composition each diet itself must conform to the first law, but because they may be carrying out different overall chemical reactions, there is no requirement that the energy changes are the same in the two biological reactions just because the reference calorimeter values are the same. In addition, it is expected that different pathways will have different efficiencies as dictated by the second law. Thus, it is not thermodynamics, but the special characteristics of living systems that explain why energy balance is usually observed. Under most conditions, a steady state can be attained in which oxidation of food to CO_2 _and water is the major process, and the differences between the diets in the other reactions are small.

Finally, as noted above, application of thermodynamic laws is limited in systems that do not come to equilibrium. This has been described in the literature as the inappropriate use of ΔG values [37] when what is really measured under conditions where equilibrium is not attained is (∂G/∂ξ)_T,P _where ξ is the reaction progress coordinate. In the end, a thorough going analysis of the potential for inefficiency must consider nonequilibrium conditions.

## Conclusion

Emphasis on kinetics and nonequilibrium thermodynamics provides a conceptual framework for understanding the effect of macronutrient composition on maintenance and change of body mass and possibly for analysis of adipocyte metabolism in general. The simple model presented is intended to be consistent with a general shift away from equilibrium thermodynamics and towards a more dynamic analysis of cellular processes.

## Abbreviations

FA: fatty acid

FIRKO: Adipose-specific insulin receptor knockout mice

LPL: lipoprotein lipase

PEPCK: phosphoenolpyruvate carboxykinase

R_a _: rate of appearance

TAG: triacylglycerol (triglycerides)

TZD: thiazolidinedione

## Competing interests

The author(s) declare that they have no competing interests.

## Authors' contributions

The authors contributed equally to the preparation of this work and have read and approved the final manuscript.
